# *Plasmodium berghei* crystalloids contain multiple LCCL proteins

**DOI:** 10.1016/j.molbiopara.2009.11.008

**Published:** 2010-03

**Authors:** Sadia Saeed, Victoria Carter, Annie Z. Tremp, Johannes T. Dessens

**Affiliations:** Department of Infectious and Tropical Diseases, London School of Hygiene & Tropical Medicine, Keppel Street, London WC1E 7HT, United Kingdom

**Keywords:** Protein trafficking, Enzyme complex, Malaria transmission

## Abstract

Malaria crystalloids are unique organelles of unknown function that are present only in the mosquito-specific ookinete and early oocyst stages of the parasite. Recently, crystalloid formation in *Plasmodium berghei* was linked to the parasite protein *Pb*SR, a member of the *Plasmodium* LCCL protein family composed of six modular multidomain proteins involved in sporozoite development and infectivity. Here, we show by fluorescent protein tagging that two other LCCL protein family members are targeted to the crystalloids in a similar way to *Pb*SR. These results extend the similarities between the LCCL proteins, and provide strong supporting evidence for the hypothesis that members of this protein family work in concert and are involved in a similar molecular process.

## Graphical abstract

This paper describes the gametocyte-specific expression and subsequent targeting of two LCCL proteins to the crystalloids of *Plasmodium berghei* ookinetes using GFP-tagging.

Transmission of malaria parasites starts with the ingestion of gametocytes by vector mosquitoes during blood feeding on a parasite-infected host. Rapid gametogenesis and fertilization occur in the mosquito midgut, giving rise to motile ookinetes that transform into oocysts following their traversal of the midgut epithelium. After an approximately two-week period of growth, mature oocysts release thousands of motile sporozoites that invade the salivary glands of the insect and subsequently enter the vertebrate host during blood feeding to initiate new malaria infections.

*Plasmodium* LCCL proteins are a family of proteins important for malaria parasite transmission. Their name is based on the ***L**imulus* clotting factor **C**, **C**och-5b2, **L**gl1 (LCCL) domain [1] that is present in all but one family member. Six family members (named *P*LAPs or *P*CCps) have been identified to date, which are predicted modular proteins containing ER signal peptides and multiple adhesive domains implicated in lipid, protein and carbohydrate binding [2–6]. All LCCL protein family members identified to date are highly conserved between *Plasmodium* species. For example, *P. falciparum* *Pf*CCp3 not only has an identical domain composition and topology to its *P. berghei* orthologue *Pb*SR (also known as *Pb*LAP1), but shares 63% and 76% amino acid identity and similarity, respectively. *Pb*SR is the founding member of the LCCL protein family and was the first to be characterized by gene disruption, which revealed an essential role in sporozoite, but not oocyst, development in mosquitoes [4]. It was shown subsequently that low levels of sporozoite formation were supported in *Pb*SR knockout parasites, but salivary gland infectivity was never observed [7]. Very similar loss-of-function phenotypes involving sporozoite development have been described for other members of the family in *P. berghei*: *Pb*LAP2, 4, 5 and 6 [8,9]. In *P. falciparum*, knockout of *Pf*CCp3 (orthologue of *Pb*SR) or *Pf*CCp2 (orthologue of *Pb*LAP4) appeared to have no adverse effect on oocyst sporulation, but the resulting sporozoites were again not infective to mosquito salivary glands [5]. The reported differences in sporulation rates between *Pb*SR and *Pf*CCp3 knockout parasites may be the result of small differences in the function of the LCCL proteins between the two malaria species. In view of the compelling structural conservation of these proteins it seems equally likely that they may reflect quantitative rather than qualitative differences that are influenced by the distinct experimental setups (such as vector species) used in these studies. The latter is supported by the fact that very different sporulation levels of *Pb*SR knockout oocysts are observed under *in vitro* and *in vivo* conditions [7]. What is clear in both species is that LCCL proteins have critical roles in the development of infective sporozoites, pointing to the oocyst as a likely site of action.

Despite their apparent roles associated with sporozoite development and infectivity, both *Pb*SR and *Pf*CCp3 are synthesized in gametocytes rather than, as one might intuitively expect, in oocysts or sporozoites [5,7,10]. Growing evidence suggests the same applies to the other LCCL proteins in *P. falciparum* [2,5,11–13] and in *P. berghei* [6,14,15]. These proteins are thus unusual in that their synthesis precedes their apparent function by several days and, for that matter, by several developmental transitions, the precise reason for which has remained unclear. Recently, it was demonstrated that *Pb*SR, after being synthesized in macrogametocytes, is trafficked to the oocysts in an unusual way, namely via the ookinete's crystalloids [7], offering an explanation for the observed gap between protein synthesis and function. Crystalloids are transient organelles resembling cytoplasmic inclusion bodies that form in ookinetes and disappear after ookinete-to-oocyst transformation. The function of the *Plasmodium* crystalloids is poorly understood, but it has been postulated that they may constitute a reservoir of protein synthesized by the macrogametocyte that is used by the parasite during oocyst growth and sporozoite development [16,17]. The recent study by Carter et al. [7] also showed that parasite lines that lacked *Pb*SR expression, or that expressed a dysfunctional mutant version of *Pb*SR, did not form crystalloids. This discovery of a functional link between the crystalloids and *Pb*SR points to a central role for the crystalloids in the functioning of *Pb*SR and, potentially, other LCCL protein family members. The similarities between LCCL protein family members with respect to their structures, expression patterns, and loss-of-function phenotypes suggests that they could be involved in the same molecular processes and could be operating in concert. To further investigate this hypothesis we decided to determine the protein expression, subcellular localization and trafficking, in live *P. berghei* parasites, of two other members of the family, *Pb*LAP2 and *Pb*LAP3 (also known as *Pb*CCp1 and *Pb*CCp5, respectively), using a green fluorescent protein (GFP) tagging approach.

*Pb*LAP2 is encoded by a single exon gene and is composed of 1614 amino acids, while the *pblap3* gene contains a single predicted intron and encodes a 1049 residue protein. Both gene products possess predicted amino terminal ER signal peptides, which upon cleavage gives rise to mature proteins of predicted sizes of 180 kDa (*Pb*LAP2) and 120 kDa (*Pb*LAP3). *Pb*LAP2 shares 67% and 80% amino acid identity and similarity, respectively, with its *P. falciparum* orthologue *Pf*CCp1. For *Pb*LAP3 and its *P. falciparum* orthologue *Pf*CCp5 these values are 52% and 69%, respectively. The domains identified in *P*LAP2 (*P*CCp1) and *P*LAP3 (*P*CCp5) have been described previously [2–6]. Briefly, the identified protein domains of *P*LAP2 (*P*CCp1) include a ricin B-related domain; a discoidin-like domain; a fibrillar collagen-associated domain; one LCCL domain; two tandem levanase-like domains; and two tandem carboxy-terminal cysteine-rich domains. The identified modules of *P*LAP3 (*P*CCp5) include besides a single LCCL domain a fibronectin-like domain, an anthrax protective antigen-like domain, and a discoidin-like domain.

To achieve GFP-tagging of *Pb*LAP2 we adopted a strategy of single crossover homologous recombination (Fig. 1A). A 2.3 kb fragment of *pblap2* corresponding to the 3′-part of the coding sequence was PCR amplified from genomic DNA with primers P1 and P2 (Fig. 1A) and introduced into *Sal*I/*Hin*dIII-digested pDNR-EGFP [7], via in-fusion cloning (BD Biosciences) to give plasmid pDNR-PbLAP2/EGFP. The *pblap2/egfp*-specific sequence was then transferred to pLP-hDHFR via cre-*lox*p recombination to give plasmid pLP-PbLAP2/EGFP (Fig. 1A). Plasmid pLP-hDHFR was previously constructed by introducing the human *dhfr* gene cassette (i.e. *hdhfr* flanked by 5′ and 3′ UTRs from *pbdhfr*) into *Sphl*I/*Nde*I-digested pLP-DHFR2 [18]. To achieve GFP-tagging of PbLAP3 we used a strategy of double crossover homologous recombination (Fig. 1B). The entire *pblap3* coding sequence plus 0.6 kb of upstream sequence was PCR amplified from genomic DNA with primers P3 and P4 (Fig. 1B) and cloned into *Sal*I/*Hin*dIII-digested pDNR-EGFP to give plasmid pDNR-PbLAP3/EGFP. The 3′ UTR of *pblap3* was amplified with primers P5 and P6 (Fig. 1B) and the resulting 0.7 kb fragment cloned into *Nde*I-digested pLP-hDHFR by in-fusion cloning to give plasmid pLP-hDHFR/PbLAP3. The *pblap3/egfp*-specific sequence from pDNR-PbLAP3/EGFP was transferred to pLP-hDHFR/PbLAP3 by cre/*lox*p recombination to give the final construct pLP-PbLAP3/EGFP (Fig. 1B).

pLP-PbLAP2/EGFP was linearized with *Pac*I prior to transfection of purified schizonts. pLP-PbLAP3/EGFP was doubly digested with *Kpn*I and *Sac*II prior to transfection (Fig. 1). After transfection, pyrimethamine-resistant parasites were selected and cloned as described [19] to give parasite lines *Pb*LAP2/EGFP and *Pb*LAP3/EGFP, respectively. Diagnostic PCR using primers P7 and P8 (Fig. 1A) amplified a unique 2.5 kb fragment from parasite line *Pb*LAP2/EGFP (Fig. 1C), confirming correct integration of the *egfp* sequence downstream of the *pblap2* allele. Diagnostic PCR using primers P9 and P10 (Fig. 1B) amplified a unique 1.8 kb fragment from parasite line *Pb*LAP3/EGFP (Fig. 1C), confirming correct integration of the *hdhfr* selectable marker gene cassette into the *pblap3* locus. Both parasite lines displayed normal parasite development in mouse and mosquito hosts, indicating that the GFP tags did not adversely affect protein function.

To study *Pb*LAP2 and *Pb*LAP3 protein expression we assessed live parasites by confocal and UV microscopy. Blood stage parasites of *Pb*LAP2/EGFP and *Pb*LAP3/EGFP parasite lines displayed green GFP-based fluorescence in gametocytes (Fig. 2A), confirming the gametocyte-specific *Pb*LAP2 and *Pb*LAP3 expression predicted from GFP reporter studies [15,20]. Both *Pb*LAP2 and *Pb*LAP3 were observed distributed throughout the parasite cytoplasm in a somewhat punctate pattern (Fig. 2A), which is very similar in appearance to the subcellular localization of *Pb*SR observed in this life stage [7]. In ookinetes, on the other hand, the typical distribution of *Pb*LAP2 and *Pb*LAP3 was confined to two focal spots, often visibly associated with clusters of malaria pigment (Fig. 2B). This localization is again very similar to that observed in ookinetes of parasite lines expressing GFP- or red fluorescent protein (RFP)-tagged *Pb*SR [7]. GFP-based fluorescence was neither observed in mature oocysts (Fig. 2C) nor in midgut- and salivary gland-associated sporozoites (data not shown), indicating that neither *Pb*LAP2 nor *Pb*LAP3 are present at discernible levels during this part of the life cycle. These observations are again in full agreement with GFP reporter studies [15]. The unique appearance of the fluorescent spots found in ookinetes of parasite lines *Pb*LAP2/EGFP and *Pb*LAP3/EGFP (Fig. 2B), in particular their co-localization with malaria pigment, strongly indicated that they correspond to the crystalloids as was recently demonstrated for *Pb*SR [7]. Indeed, the presence of *Pb*LAP2 and *Pb*LAP3 in crystalloids was confirmed by immunogold EM experiments (Fig. 2D) carried out as previously described [7]. Thus, the expression pattern, subcellular localization and trafficking of *Pb*LAP2 and *Pb*LAP3 appear to be very similar, if not identical, to those of *Pb*SR [7].

Our data demonstrate the use of GFP-tagging of *Pb*LAP2 and *Pb*LAP3 in genetically modified *P. berghei* lines to determine their expression, subcellular localization and trafficking in live parasites. Our data show that *Pb*LAP2 and *Pb*LAP3 are both targeted to the crystalloids, similar to *Pb*SR, thereby increasing by three-fold the total number of crystalloid proteins identified to date. Within the LCCL protein family, *Pb*LAP2 and *Pb*LAP4 are close structural paralogues, and the same is true for *Pb*LAP3 and *Pb*LAP5. It is quite likely, therefore, that *Pb*LAP4 and *Pb*LAP5 display the same expression and trafficking as shown here for their structural paralogues. The fact that three structurally distinct LCCL protein family members follow the same unusual protein trafficking pathway in *P. berghei* adds strong experimental support for the hypothesis that at least several, and perhaps all, *Plasmodium* LCCL proteins are involved in the same molecular processes facilitating sporozoite development and infectivity. It is likely that this is achieved as a molecular complex containing multiple LCCL protein family members. Indeed, evidence for intermolecular interactions of different LCCL proteins in *P. falciparum* gametocytes was recently reported [13].

Using a different detection method, indirect immunofluorescence, *Pf*CCp molecules in *P. falciparum* gametocytes have been shown to associate with the parasite plasma membrane, parasitophorous vacuole, and even the host erythrocyte [5,10–13], suggesting an extracellular role for these molecules during gametogenesis and fertilization. We did not observe clear evidence for a similar scenario in *P. berghei* (for instance, an accumulation of the LCCL proteins at the parasite periphery as observed in *P. falciparum*), but this could reflect the substantial differences in gametocytogenesis that exist between the two *Plasmodium* species. The observed pattern of distribution of *Pb*LAP2 and *Pb*LAP3 (Fig. 2A) and, for that matter, *Pb*SR [7] in gametocytes is, in fact, not inconsistent with a vesicular localization, which could point to secretion of these proteins during gametogenesis as appears to be the case in *P. falciparum*. Thus, while it is clear that after fertilization *Pb*SR, *Pb*LAP2 and *Pb*LAP3 redistribute to the crystalloids, we should consider the possibility that some of the protein may be secreted before this event.

This paper provides further evidence that the crystalloids play a central role in the function of the LCCL proteins in *P. berghei*. The exact nature, however, of the molecular processes that lead to crystalloid formation to facilitate sporozoite development and infectivity remains poorly understood. The two new cellular markers for the crystalloids identified here will provide useful new tools for addressing this intriguing question.

## Figures and Tables

**Fig. 1:**
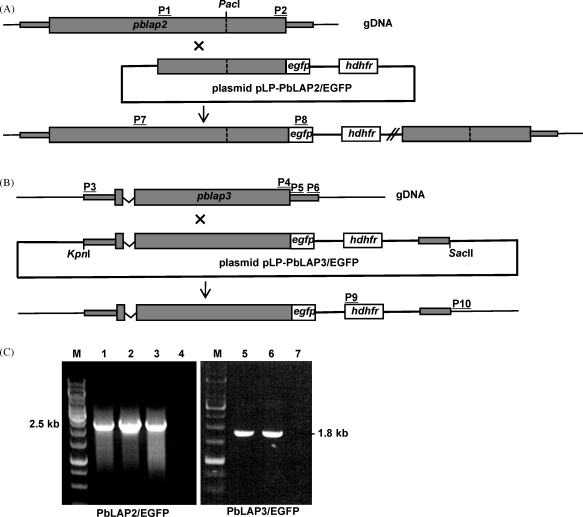
Generation and molecular analysis of genetically modified parasite lines. (A) Targeting strategy for GFP-tagging *Pb*LAP2 via single crossover homologous recombination. (B) Targeting strategy for GFP-tagging *Pb*LAP3 via double crossover homologous recombination. The *pblap* genes are indicated with coding sequence (wide bars) and noncoding sequence (narrow bars). Also indicated are the enhanced GFP module (e*gfp*); the human *dhfr* selectable marker gene cassette (*hdhfr*); the intron in *pblap3* (v-shaped line); the position of key restriction sites (*Pac*I, *Kpn*I, *Sac*II); and primers used for PCR amplification (P1–P10). (C) Diagnostic PCR of genomic DNA from three PbLAP2/EGFP clones (lanes 1–3) and two PbLAP3/EGFP clones (lanes 5 and 6). In lanes 4 and 7 genomic DNA (gDNA) from wild-type parasites was used as template. M = Generuler 1 kb DNA ladder (Fermentas).Primer sequences: P1(ACGAAGTTATCAGTCGACATGAGTCATTACTAGACATAATTACAAGTGAA); P2(ATGAGGGCCCCTAAGCTTTCAGTAATTCCATGAGTTACTTTGC); P3(ACGAAGTTATCAGTCGAGGTACCTAGCGGAAACAACAATGTTC); P4(ATGAGGGCCCCTAAGCTATTTTTAATAATTTGTATCGAAAGTATAGTTG); P5(CCTTCAATTTCGACATATAATGGATTAAAATTTTAGTTCGGT); P6(GCGGCCGCTCTAGCATAGGATTAGAAATACAGTAATAGCAATTTTG); P7(CATCTATACATGCAGGCG); P8(GTGCCCATTAACATCACC); P9(ACAAAGAATTCATGGTTGGTTCGCTAAACT); P10 (CCTCAAGATAGTTACGAATTTAAC).

**Fig. 2:**
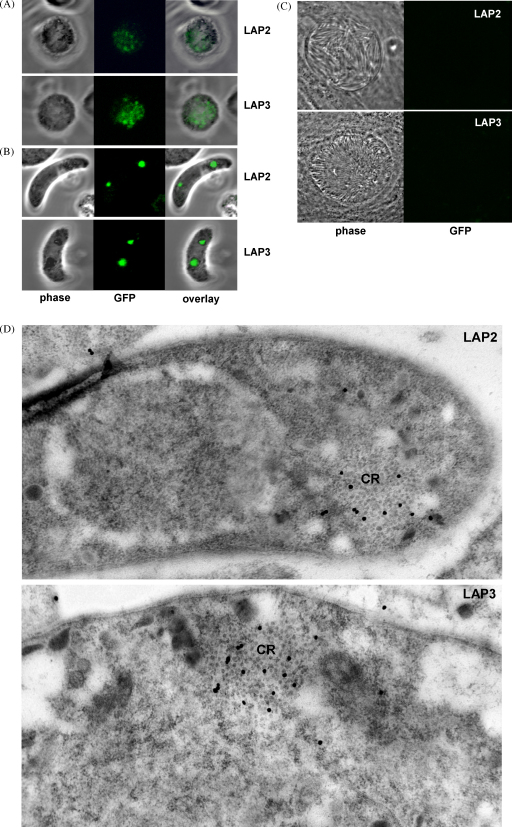
Expression and localization of GFP-tagged *Pb*LAP2 and *Pb*LAP3. (A) Confocal images of live gametocytes. (B) Confocal images of live ookinetes. (C) Confocal images of mature oocysts containing sporozoites. (D) Immunogold EM images (with silver enhancement) of purified ookinetes, showing labelling of crystalloids (CR).
